# The Adaptive Test of Emotion Knowledge for 3-to 9-Year-Olds: Psychometric Properties and Validity

**DOI:** 10.3389/fpsyt.2022.901304

**Published:** 2022-07-08

**Authors:** Katharina Voltmer, Maria von Salisch

**Affiliations:** Institute of Psychology, Leuphana University Lüneburg, Lüneburg, Germany

**Keywords:** emotion knowledge, children, adaptive test, development, measure, emotion understanding

## Abstract

Children with an advanced knowledge of emotions are generally more socially competent, less likely to suffer from psychopathology, and more likely to succeed in school, both socially and academically. The assessment of children's emotion knowledge has thus gained importance in recent decades - both in psychiatric practice and in developmental and educational psychology. However, there is still a lack of appropriate instruments for assessing children's emotion knowledge in a performance test reliably, and for a broad age range. The Adaptive Test of Emotion Knowledge (ATEM 3–9) is a newly developed measure which encompasses seven components of emotion knowledge in 3–9-year-olds. The ATEM 3–9 is an adaptive test which uses skip and dropout rules to adjust for children's varying levels of knowledge. In addition to German, the ATEM has been translated into English and Hebrew. The German norming sample of the ATEM 3–9 comprises *N* = 882 (54% female, 21% bilingual) children between the ages of 3 and 9 years, who were divided into seven age groups. Test items, which are ordered according to the item response theory, showed a good fit to a seven-dimensional model reflecting the seven components. The internal consistencies of the dimensions are acceptable to good. Construct validity was examined by means of correlations with other measures of emotion knowledge, as well as measures on language skills and executive functions in a subsample. This resulted in medium size correlations in the expected directions. In addition, children with externalizing and internalizing disorders who were recruited in psychiatric in- and outpatient clinics showed deficits in various components of emotion knowledge when compared to their agemates in the norming sample. Overall, the ATEM 3–9 is well suited to measure individual components of emotion knowledge in children and to obtain a differentiated picture of the various aspects of emotion knowledge. The ATEM 3–9 thus supports the investigation of the development of social-emotional competencies in normative development (e.g., school readiness) and in social-emotional-learning interventions. Furthermore, it is suitable as an instrument for the differentiated assessment of (progress of) children's emotion knowledge in clinical child psychology and psychiatry.

## Introduction

Emotion knowledge, also referred to as emotion understanding, is an integral part of emotional competencies along with emotion regulation and emotion expression. Emotion knowledge is thus a cornerstone of emotional functioning which is described, for example, in the conceptual framework of Emotion Understanding in Recognition and Knowledge Abilities (EUReKA) by Castro et al. (1). These authors use the term *emotion understanding* to refer to the recognition of and knowledge about emotions and emotional cues in oneself and others. Pons et al. (2) use the term *emotion knowledge* to describe the development of emotion recognition and knowledge about emotions in others in early and middle childhood. Thus, the two models encompass overlapping but not identical skills and competencies. Both research teams agree that emotional competencies develop in early to middle childhood and are further refined in adolescence and adulthood. Following Pons et al. (2), the present work focuses on the development and assessment of the recognition and knowledge of other peoples' emotions during early and middle childhood. In the present paper, we will call these components emotion knowledge. The present work presents results on the psychometric properties, validity, and applications of the Adaptive Test of Emotion Knowledge for 3- to 9-year-old children (ATEM 3–9) in a German sample. The ATEM 3–9 encompasses seven of the emotion knowledge components described in Pons et al. (2), which are (1) facial recognition, (2) situational recognition, (3) knowledge about desire-related emotion, (4) recognition of mixed emotions, (5) knowledge about belief-related emotions, (6) knowledge about display rules, and (7) knowledge about emotion regulation strategies. The development of these components is presented in a more detailed way in the next section. In the ATEM 3–9 the corresponding skills are measured in the order in which they develop in childhood. In this way, it is possible to verify whether the developmental course of emotion knowledge is age appropriate in typically developing children and in children who are under psychiatric treatment. Of course, other development variables also play a role in this assessment. Therefore, an overview of cognitive and social as well as sociodemographic and psychopathological correlates of emotion knowledge is provided below. Before describing the ATEM 3–9 itself in detail and pointing out the aims of the present work, a brief overview of existing emotion knowledge assessment instruments is given.

### Development of Emotion Knowledge Components

The ability to distinguish the different facial expressions of others starts in the newborn period and refines within the second and third year of life. Most 3-year-olds can read the four basic emotions of joy/happiness, sadness, fear, and anger in the faces of others (2). They are also able to label them – a competence that goes along with language development (3). Using situational cues to identify one emotion (or several emotions at the same time) starts in the fourth year of life and goes on until late childhood (2, 4). Younger children find it easiest to recognize happiness (or joy) in prototypical situations. With age, various negative emotions, such as anger, sadness, or fear, are increasingly recognized and emotions in non-prototypical situations are labeled with increasing accuracy (5). During the same period, the internal states of other people are increasingly used to identify their emotions. Theory of Mind skills are essential for identifying emotions as a function of the other person's preferences, desires, or beliefs (2, 6). Theory of Mind also plays a role when differentiating between the emotions which individuals show to others and the emotions they actually feel. The ability to recognize and understand these display rules begins around age four and is largely developed by age 6–8 years of age (7). Finally, knowledge about socially accepted, adaptive, and functional strategies of emotion regulation develop from the age of four onward. First, behavioral regulation strategies and later cognitive regulation strategies are recognized and labeled (8). In Table 1, the development of the components of emotion knowledge is summarized.

**Table 1:** Overview of emotion knowledge components and their development in early childhood.

**Component (age of development)**	**Description**
Facial recognition (2–3 years)	Recognizing emotions based on facial expressions. Often, the four basic emotions of joy, sadness, fear, and anger are given in photographs or graphic representations of children. Most 3-year-old children can reliably recognize the emotions and point to a target emotion. Labeling the emotions follows a little later.
Situational cues (3–5 years)	Situations, often presented in the form of short vignettes, typically lead to a particular emotion. By the age of about four, most children can reliably recognize the emotions of others from descriptions of typical situations.
Internal cues (3–6 years)	The (non-)fulfillment of a wish leads to particular emotions. Depending on whether the child has the same wish as, for example, the protagonist in a story, it is easier or more difficult for the child to recognize the other person's (or character's) emotion when the wish is not granted. Different beliefs lead to different evaluations of situations and thus to different emotions. It is more challenging for children to assign the correct emotions when other people hold false beliefs.
Mixed emotions (4–8 years)	People often feel two or more emotions at the same time. Recognizing mixed emotions in other people is easier when the emotions are of similar valence and more difficult when the emotions are of different valence.
Display rules (4–8 years)	Emotional expressions are subject to many social and cultural rules. Therefore, the expression and the experience of an emotion often diverge. Younger children usually recognize these differences only when they are pointed out to them. Beginning at the age of about 6 years, many children know about this differentiation, even when it is implicit.
Emotion regulation strategies (4–8 years)	From preschool age onward, children learn to regulate their emotions themselves. As they get older, they learn which strategies are appropriate in certain situations and which are not. For the most part, children first know that they can act to regulate emotions; later, they also acquire mental regulation strategies.

### Emotion Knowledge, Language Skills, and Executive Functions

Language skills and executive functions are often considered important cognitive correlates of emotion knowledge. Because emotion knowledge is mainly taught and learnt *via* emotion talk, i.e., language input during verbal interactions, receptive and expressive language competencies are strong positive correlates, especially in early childhood (3, 9, 10). Conte et al. (11) found a zero-order correlation of *r* = 0.66 between 2- and 3-year-old children's receptive vocabulary and their emotion knowledge. In a slightly older sample, the mean correlation between receptive vocabulary and emotion knowledge was *r* = 0.51 (12). Brock et al. (13) reported a zero-order correlation of *r* = 0.40 between emotion knowledge and expressive vocabulary in 5-year-olds. These studies suggest that language skills are very important when acquiring the basic knowledge about emotions before entering formal schooling. The importance of verbal abilities is underlined by studies that indicate that language skills have a positive impact on the growth trajectory of children's knowledge about emotions (10, 14, 15). Studies on children with language impairments which found deficits in children's emotion knowledge (16) further emphasize the importance of emotion talk for the acquisition of emotion knowledge (17). Better language skills thus seem to help children in acquiring emotion knowledge. However, associations seem to be bidirectional, because other studies show that language skills were predicted by emotion knowledge in 3–8-year-olds, independent of age and gender (12, 18). As Ogren and Johnson [(19), p. 113] conclude, “[…] there is an important relation between a child's language abilities and emotion understanding, but the precise mechanism behind this existing relation remains unclear.”

Just like language, executive functions cannot be separated from emotion knowledge. Oftentimes all three variables interact. A recent study suggests that children's executive functions play a role in linking their emotion knowledge and their social competence *via* verbal skills, namely by moderating the mediating effect of language skills on the relation (20). This means that the more developed the children's executive functions were, the higher was the mediating function of verbal skills on the relation between emotion knowledge and social competence. In another study, verbal skills mediated the relation between cognitive flexibility and emotion knowledge (15). The zero-order correlations between emotion knowledge and working memory and emotion knowledge and cognitive flexibility (as a component parts of executive functions) were small to medium sized (15, 20). Moreover, executive functions work as predictors of emotion knowledge above and beyond the effects of language abilities: Set shifting predicted emotion knowledge over and above mothers' age, and children's IQ, language ability, and Theory of Mind in a sample of *N* = 75 preschool children (21). Working memory also played an explanatory role in the age-related growth of emotion knowledge (22). In a longitudinal design, executive functions contributed to the prediction of the emotion knowledge of *N* = 306 3-year-old children over a period of 18 months (23). The assumption that emotion knowledge and executive functions mutually reinforce each other seems to be rational, because other studies concluded that emotion knowledge longitudinally predicted the growth of executive functions (24). There is even evidence for bidirectional effects (25). In summary, language abilities and executive functions seem to be intertwined with the development of emotion knowledge.

### Emotion Knowledge and School Success

Executive functions and language skills are well-known cognitive predictors of children's success in school. However, social skills that are associated with emotion knowledge are often forgotten as predictors of school success, even though they play a critical role in academic achievement and social success. In Denham and Brown's (26) model of social-emotional learning (SEL), children's effective social interactions are based on five social and emotional skills: (1) self-awareness, (2) self-regulation, (3) social awareness, (4) responsible decision making, and (5) relationship skills. Social awareness relies heavily on emotion knowledge. When children can recognize and understand the emotions of their classmates and teachers, i.e., when they are aware of their social partners' emotions, successful interactions are more likely. Socially aware children tend to be better liked by their classmates in school, find friends easier (27, 28), and are thus in a better position to share knowledge and other academic resources with their peers. Children with supportive relationships with teachers and peers are likely to be more engaged in school, have more personal resources available for learning, and demonstrate better academic performance (29–31). In a meta-analysis, Trentacosta and Fine (32) reported a mean association of *r* = 0.22 between emotion knowledge and social skills in children and youths. More recent studies corroborate the association between emotion knowledge and various social skills in different age-groups (33, 34). The association between emotion knowledge and academic achievements was mediated by the social behavior of school-aged children (35). Social behavior may be an explanation for the medium sized (*r* = 0.32) association found in a meta-analysis between emotion knowledge and academic achievement in children aged 3–12 years (36). A longitudinal analysis, which used an earlier version of the ATEM 3–9, found a significant influence of kindergartners' emotion knowledge on their phonological awareness just before they entered formal schooling about 1 year later (37). Taken together, many studies agree that children's emotion knowledge is, among others, an important predictor of their academic and social success in school.

### Emotion Knowledge and Psychopathology

Emotion knowledge often comes into play when children's development deviates from the typical course, i.e., in psychopathology. The most intensely researched component of emotion knowledge regarding psychopathology is emotion recognition. Evidence shows that emotion recognition in children and adolescents is impaired in many disorders like schizophrenia, mood disorders, anxiety disorders, attention deficit (hyperactivity) disorder, eating disorders, and conduct disorders with participants being less accurate in emotion recognition in general or showing biases toward specific emotions (38). Emotion recognition and emotion knowledge in general take on a special significance in autism spectrum disorders. In the late 1980s Baron-Cohen started to investigate the Theory of Mind of children with autism spectrum disorders (39) and soon began to examine children's recognition and understanding of emotions (40, 41). Studies agree that children with autism spectrum disorders often present less developed emotion knowledge than typically developing children, at least in some components. This also holds for normally intelligent or high functioning children with autism spectrum disorders [(42); for short review about emotion recognition skills see (43)]. Various interventions were developed to teach autistic children about the different components of emotion knowledge (44–47). In recent years, underlying mechanisms and moderators of this association came into the focus of research (48, 49).

In order to elucidate the emotional precursors of psychopathology in children, researchers have oftentimes investigated the associations between emotion knowledge and self-reported or third-party reported internalizing and externalizing problem behaviors. A meta-analysis established small correlations between emotion knowledge and concurrent or longitudinal internalizing and externalizing behavioral problems (both *r* = −0.17) without any direction of effect (32). Negative correlations of a similar magnitude were found in later studies by Tarullo et al. (50) and Martin et al. (51). Correlations were higher for children with post-traumatic stress disorder and obsessive-compulsive disorder in a study of clinically anxious children (52). In a cross-sectional regression design with *N* = 87 6- to 10-year-olds, Dede et al. (53) showed that facial emotion recognition skills for fear predicted internalizing symptoms. Longitudinal designs which support a causal interpretation of these associations are rare, however, even though they highlight the predictive and preventive power of emotion knowledge for (later) behavioral problems (54–56). However, Göbel et al. (57) only found small relations of emotion knowledge with internalizing problems and no significant cross-sectional associations with externalizing problems.

### Emotion Knowledge and Sociodemographics

Given that emotion knowledge differs widely among children of different age groups, research evidence regarding gender differences is not entirely clear. While studies agree that boys and girls do not tend to differ in their overall or general knowledge of emotions (11, 24, 58–61), studies of individual components sometimes result in differences in favor of girls. Girls seem to be better able to differentiate between situational causes for emotions (62–66), to better understand mixed emotions (4), and to know more about internal causes of emotions (60) than boys. A small but relevant female advantage on the processing of emotional expressions on the face was discovered in a meta-analytic review of studies including infants, children, and adolescents (67). One explanation for these findings may lie in differences in the socialization of emotions in boys and girls. Because girls are more frequently engaged in conversations about emotions than boys, they are more likely to be encouraged to recognize emotions in others (62, 68).

Some studies take into account the socioeconomic background of the children's families and specialize in the emotion knowledge of children from low-income families (69–72). Studies agree that, on average, children from less privileged households tend to display less emotion knowledge than children from middle class families (59, 73–75). Children from immigrant families are particularly likely to live in homes with a low socioeconomic status. This may be another reason – in addition to the reduced language skills described above – for their oftentimes lower knowledge of emotions. Indeed, even in studies in which receptive language skills were controlled for or emotion knowledge was assessed in the children's native language, differences in performance were found between children with and without an immigrant background (76–79). Differences in the rules of when and how emotions are to be expressed and talked about as well as different socialization processes within families of children from different cultures, offer further conclusive explanations for these differences (80–82). Children from immigrant families do not only have to acquire typical situational causes and expression and feeling rules in their own culture, but they also have to learn these rules in the majority culture (83).

### Instruments for Assessing Emotion Knowledge

An instrument for assessing emotion knowledge in children should encompass the components described above, while taking into account the developmental course of emotion knowledge and associations with children's cognitive abilities. The instrument should cover as many components as necessary, or as expected to be solved in a particular age-group, with as few demands on children's language abilities and attention and memory skills as possible. In addition, it should be designed to be time-efficient and attractive for children. Apart from the many studies in which are confined to the recognition of emotions in children's faces [recently also with dynamically changing facial expressions (84)], two types of tasks were given in most studies which assessed children's knowledge of the basic emotions of happiness/joy, sadness, fear, and anger: First, children were asked to recognize emotions in the facial expressions of people in photographs or drawings; second, pictures of stories were presented or acted out with hand puppets, in which children were asked to recognize the emotions that the situations typically evoke (1). Thus, the two components that most children acquire between the ages of three and four (i.e., facial recognition and situational recognition of basic emotions) were mainly measured in this way. These tests are independent from expressive language skills because children point at the facial expressions of emotions and are not asked to name the emotions. The Affective Knowledge Test (85) and the Emotion Matching Task (86, 87) are well known instruments that contain these tasks. Additionally, these instruments include language-dependent emotion naming tasks. The Emotion Understanding Assessment (88) includes items to assess the knowledge of desire-based and belief-based emotions and is thus an exception, because mostly study-specific instruments have been developed for measuring the more complex components of emotion knowledge which are connected with children's Theory of Mind. Recognizing that emotions can result from false beliefs or separating the experience from the expression of emotion (which slightly older children can solve), are examples for these more sophisticated components of emotion knowledge (14). A rare exception is the Test of Emotion Comprehension (TEC) (89) which comprises nine components of emotion knowledge. The TEC uses one (mixed emotions, moral emotions, emotion regulation strategies, display rules) to five (emotion recognition and situation knowledge) short vignettes with line-drawn pictures to assess each component. The advantages of the TEC are that it examines the nine components within in a short period of time and that it can be used for children between the ages of 3 and 10. Expressive language skills are not required in the TEC. To prevent possible gender effects, it is available in a boy's and a girl's version. A major drawback is that the TEC covers only the two emotion recognition components (facial recognition and situational recognition) with five items each. Because the more demanding components are recorded with only one or two items per component, these components can only be asked for regarding one (or two) target emotions. Moreover, there is a 25% probability of correctly solving a one-item-component by guessing. Interpreting emotion knowledge at the emotion and the component levels is therefore not possible with the TEC. Instead, the components of the TEC form three basic levels of emotion knowledge which are arranged in a hierarchical order: (1) The external aspects are composed of emotion recognition abilities (facial, situational, mixed emotions) and develop before entering school at around the age of six. (2) The mental aspects are understanding emotions resulting from desires, beliefs, and memory and develop between the ages of five and seven. (3) And the reflective aspects are knowledge about emotion regulation strategies, display rules, and moral emotions that develops between the ages of seven and nine. Younger children are asked to complete the entire test, although they are usually not yet able to answer a large part of the items correctly. This may lead to frustration.

### The ATEM 3–9

With the ATEM 3–9, a test has been developed that captures seven components of emotion knowledge in early and middle childhood. According to the rules of Item Response Theory (IRT), the components are arranged in a way which reflects the developmental course of the individual competencies over time, i.e., items within and between components increase in difficulty. This makes it possible to give a child only a subset of the available items within the components, and to do so until the child's ability limit is reached. It also allows to present to the child only a subset of the available components when it is foreseeable that the more difficult components can no longer be solved. The components of emotion knowledge in the ATEM 3–9 (Table 2) can be assessed without expressive language skills. Children are asked to read facial expressions of emotions, to identify external and internal reasons for emotions, and to distinguish between shown and felt emotions and between adaptive and maladaptive strategies of regulating emotions. In addition to the basic emotions of happiness/joy, sadness, fear, and anger, ATEM 3–9 includes items on surprise and disgust. The ATEM 3–9 is adaptively designed by means of skip and termination rules within and between the components. The ATEM 3–9 is structured and looks like a picture book that tells a story about two children who visit a zoo with their parents. Each setting in the story corresponds to a particular component of emotion knowledge (e.g., Component 6: Petting Zoo). When a component is completed, the setting in the story also changes. Components 1 and 2 each include six items, components 3 through 6 each include five items, and component 7 includes three items. ATEM 3–9 is available in a girl's version and a boy's version with two female or two male characters, respectively. The names and the drawings suggest an immigrant background for one of the two characters in the story. In a one-to-one setting, the ATEM 3–9 can be completed both on paper and in an electronic offline (Android application) or online (test-platform) version with the story as an audio output on a tablet or a computer. The digital version opens up the possibility to complete the ATEM 3–9 over long distances with video conference tools.

**Table 2:** Components of the ATEM 3–9.

**Component (no. of items)**	**Task**	**Story**	**Item example**
Facial recognition (6)	Recognize emotions based on facial expressions	The characters of the story are introduced	This is Katie. Katie is usually happy. Can you show me what Katie looks like when she is happy?
Situational recognition (6)	Recognize emotions based on situation descriptions with and without distractors	The characters pack their things for the zoo and go by train	Katie's mother doesn't allow Katie to wear her new pants at the zoo. Can you show me how Katie feels when her mother doesn't allow her to wear her new pants?
Desires (5)	Recognize emotions by the (non-) fulfillment of wishes. The story characters' wishes can correspond to those of the child, or be contrary to them	Characters visit the elephants (characters' wishes match those of the child) and the monkeys (characters' wishes do not match those of the child)	Katie and Selena also want to sit on the elephant's trunk and be carried. And the animal keeper lets them. Can you show me how Katie and Selena feel when they are allowed to sit on the elephant's trunk?
Mixed emotions (5)	Recognize mixed emotions of same and different valence	The characters wait outside a gift store (same valence) and have a discussion with the parents (different valence)	Katie and Selena get money from Katie's parents to spend in the zoo. Katie and Selena want to buy lots of candy with the money. But Katie's mother forbids Katie to spend all the money on candy. Can you show me how Katie feels when she gets money, but her mother forbids her to spend it all on candy?
Beliefs (5)	Recognize emotions from different and from false beliefs	Characters visit the birdhouse (different beliefs) and a restaurant (false beliefs)	For dessert Katie's mother ordered a hot chocolate. She says Katie is allowed to taste. But Katie thinks the hot chocolate is much too hot and she will burn her tongue. But actually, the hot chocolate isn't hot anymore. Can you show me how Katie feels when she's expected to try the hot chocolate?
Display rules (5)	Distinguish shown and expressed emotions	Characters visit the aquarium (recognize only felt emotions) and the petting zoo (recognize both expressed and felt emotions)	Selena sees another friend from school at the snake enclosure. She wants to look at the snakes with Selena. But Selena would much rather look at the snakes with Katie and not with her other friend. Selena doesn't want to hurt her other friend's feelings and looks at the snakes with her although she would much rather do that with Katie. But the look on her face is therefore different than what she really feels. Here you can see what her face looks like. She looks happy. Can you show me how Selena really feels inside?
Emotion regulation strategies (3)	Recognize functional emotion regulation strategies of a behavioral (remove stimulus) and cognitive (distraction and reappraisal) nature	Follow-up items on two items of component 2 and one item of component 6	Selena is angry because Katie cut in line. What could Selena best do to stop feeling angry? 1. Selena could also cut in line 2. Selena could think that Katie is always allowed to do things first 3. Selena can't do anything to stop being angry 4. Selena could think that she can still touch the fish

The aim of the present paper is to describe the factorial structure and estimates of reliability and validity of the ATEM 3–9, as well as the psychometric properties of the items. Multidimensional item response theory (MIRT) makes it possible to analyze the multidimensional construct emotion knowledge. Therefore, a seven-dimensional model of emotion knowledge representing the seven components of the ATEM 3–9 is compared to a one-dimensional solution, with (1) an expected superiority of the seven-dimensional model. Because the ATEM 3–9 is designed to reflect the development of emotion knowledge in childhood, children's performances are (2) expected to increase with age. The gender-specific versions of the test may facilitate the identification of girls and boys with the characters in the test and thus prevent effects of gender due to misidentification. However, because some studies found that girls tend to perform better in some aspects of emotion knowledge, we (3) expect higher scores for girls than for boys in the ATEM 3–9. (4) Multilingual children are expected to score lower on the ATEM 3–9, especially in the more complex components with high text load because of the effects of receptive language skills. Correlations of the ATEM 3–9 score with language skills, executive functions, and other measures of emotion knowledge are described as indicators of construct validity. (5) We expect medium-size positive correlations with the other emotion knowledge measure (TEC) and with the measures of language skills and executive functions, because of overlapping but not identical constructs of emotion knowledge and because of the complex interrelations between language skills, behavioral self-regulation, and emotion knowledge. Differences in emotion knowledge between children with and without mental disorders are considered evidence of criterion validity. (6) Based on the literature on emotion knowledge and psychopathology, typically developing children are expected to score higher on the ATEM 3–9 than their agemates with a diagnosed mental disorder. A last aim of the present paper is to make some suggestions for the application of the ATEM 3–9 in research and (clinical) practice.

## Methods

### Sample and Procedure

Analyses are based on the norm sample of the German version of the ATEM 3–9. In total, the norm sample included *N* = 882 children between the ages of three and nine (mean age = 72.13 months, SD = 21.17), from whom data were collected between June 2019 and September 2020. Overall, 54% of the children were female. The proportion of children growing up in multilingual households ranged from 16% to 33% percent, depending on the age group. Children from kindergartens and primary schools located in Lower Saxony, Hamburg, and two other German cities participated. Data on the socioeconomic status of the children's families was not available. Children were tested individually using the digital version of the ATEM 3–9 on tablets with an audio output. The tablet was usually placed on a table and was initially operated by the test leaders. Through the audio output, both the instructions of the test were given, and each test item was read aloud. The children themselves only had to tap on one of the four given emotion pictures to select their solution. Thus, the independent execution of the test with the tablet was possible even for 3-year-olds. Children's data entry was supported, only if necessary, by the test leaders who were always present. For a proportion of 34% of the children, the test ended after the second component due to the termination rule. Most of these children (85%) were younger than 6 years of age. When individual components (because of the skipping rules) were abbreviated or the whole test was terminated after component 2, it took less time to finish the ATEM 3–9. The mean processing time for children between 3 and 5 years of age (preschoolers) was 15 min, and for children between 6 and 9 years (school-aged children) it was 25 min. A subsample of *n* = 160 children (of 3–6 years of age) was also tested individually on sentence comprehension, and another subsample of *n* = 48 children between 6 and 9 years of age was also tested individually on receptive vocabulary. A subsample of *n* = 220 children between the ages of three to six was additionally tested on behavioral self-regulation. These additional tests extended the interview by about 20 min for these children. Only children for whom parental consent form was available and who had voluntarily agreed to participate, took part in the data collection. Table 3 provides an overview over the composition of the norm sample.

**Table 3:** Sample characteristics of the norm sample of the ATEM 3–9.

**Age (years)**	***N* (%)**	**Gender (%)**		**Language (%)**	
		**Male**	**Female**	**German**	**Multilingual**
3	120 (13.60)	59 (49.17)	61 (50.83)	99 (82.50)	21 (17.50)
4	131 (14.85)	60 (45.80)	71 (54.20)	110 (83.97)	21 (16.03)
5	175 (19.84)	76 (43.43)	99 (56.57)	124 (70.86)	51 (29.14)
6	160 (18.14)	71 (44.38)	89 (55.62)	132 (82.50)	28 (17.50)
7	79 (8.69)	37 (46.84)	42 (53.16)	53 (67.09)	26 (32.91)
8	143 (16.21)	67 (46.85)	76 (53.15)	121 (84.62)	22 (15.38)
9	74 (8.39)	40 (54.05)	34 (45.95)	58 (78.38)	16 (21.62)
Total	882 (100)	410 (46.49)	472 (53.51)	697 (79.02)	185 (20.98)

In addition to the norm sample, a sample of *N* = 55 children between the ages of six and nine (mean age = 99.1 months, SD = 12.64; 36% female) was included. These children had been diagnosed with at least one mental disorder by means of test psychology by a licensed therapist in child and adolescent psychiatry or psychotherapy. Of these children, 44% presented an internalizing disorder and 25% an externalizing disorder. Eighteen percent of the children had a mixed disorder, while another 13% were diagnosed with other disorders.

An in-depth study was conducted with an earlier paper-pencil version of the ATEM 3–9 with 40 items in the intervention project “Feeling Thinking Talking” which was designed to promote children's language skills, emotion knowledge and scientific thinking by training kindergarten teachers to use child-adapted language promotion strategies and emotion talk in everyday situations (FTT) (90, 91). At the first measurement point of the FTT study, the final 32 items of components 1–6 of the ATEM 3–9 and the resulting total test score were evaluated in *N* = 211 3- to 5-year-olds (mean age = 50 months, SD = 7.2). Of these children, 47% were female and 20% grew up in multilingual households according to parent report. Unfortunately, the information on multilingualism was missing for 33% of the children. Combined teacher and parent reports agree that for 43% of the children in the FTT sample at least one parent was born abroad. Among others, the FTT assessments included individual assessments of receptive and expressive vocabulary and behavioral self-regulation, as well as additional measures of emotion knowledge.

### Materials

#### ATEM 3–9

Developing the final version of the ATEM 3–9 took several steps. Initially, the item pool for the ATEM included 100 items, which were divided among six components of emotion knowledge, six distinct emotions (joy, fear, sadness, anger, disgust, and surprise), and two distinct test versions (with 12 anchor items and 44 parallel items each). The seventh component (emotion regulation strategies) was added in a later step. After a first pilot study with these 100 items, six anchor-items, which were too easy, and 44 items with poorer item characteristics than the corresponding item in the parallel version were excluded, resulting in a 50-item first version of the ATEM 3–9. In addition, skipping and termination rules were developed to simplify the use with young children. The first rule mandates that all children perform component 1 and 2 but stop the test after component 2, if they fail on three items in a row within these two components. The second rule mandates that children skip the following items of components 3 through 6 and start the following component (stop the test at component 6), if they fail to solve three items in a row in the respective component. This first test version turned out to be too long for preschool children. It also contained items on both positive and negative surprise, which would have led to inconsistencies. Therefore, items about negative surprise and items with poorer psychometric characteristics were excluded from the test, resulting in a second version with 40 items within six components. This version was used in the FTT intervention project. It was still too long for many children, resulting in dropping additional items with poorer item characteristics in each component, leading to a 32-item version. Acknowledging the importance of strategies of emotion regulation, three items on this component were added in a last step. These items ask about three different types of functional emotion regulation, i.e., problem-oriented behavior, attention shifting, and reappraisal. The three items of this component are located within component 2 and component 6. Termination and skipping rules do not apply to these items. Simple items in this component ask about emotion regulation strategies that are easy to apply (problem-oriented behavior, attention shifting) within the component 2. The more difficult regulation strategy (reappraisal) is presented at the end of the test among the items of component 6. In the final 35-items version of the ATEM 3–9 the first and second component contain six items and the third to sixth component consist of five items with the target emotions happiness/joy, (positive) surprise, anger, sadness, fear, and disgust. Component 7 consists of three items which target strategies for regulating fear, anger, and disgust. The target emotion of each item is presented in **Table 5**. The scores for each component and the total score were obtained by summing the correct answers for each component and for the total test.

#### Instruments for Validation

Receptive vocabulary was assessed both in a subsample of the norm sample and in the FTT sample with the German version of the Peabody Picture Vocabulary Test (PPVT-4) (92). The PPVT-4 was designed for children between the ages of three and sixteen and consists of 228 items organized in an adaptive structure with age-related starting points as well as reversal and termination rules. The children are presented items consisting of four pictures. They are then asked to point to the picture which corresponds to the word named by the experimenter (“Point to...”). Thus, children can score a maximum of 228 points. The internal consistency of the PPVT-4 was very high in the FTT sample with Cronbach's α = 0.98.

The subtest Sentence Understanding of the German Language Development Test for 3- to 5-year-old children (SETK 3–5) (93) was used to assess sentence comprehension in a subsample of the norm sample and in the FTT sample. Children score points for carrying out the experimenter's instructions with various materials. The test contains 15 sentences of increasing grammatical complexity, resulting in the highest score of 15 points. In the norm sample Cronbach's α was 0.87 and in the FTT sample it was 0.88.

In the FTT sample, the Active Vocabulary Test-Revised (AWST-R) (94) was used to assess children's expressive vocabulary. It is suitable for children between the ages of three and five and a half years. The children are presented a series of cards on which objects or activities are depicted. For the 51 nouns, the interviewer asks the question “What is this?” and for the 24 verbs, “What does he/she do?” The test is terminated if the children cannot solve any of the first 10 items correctly. Correct answers yield the raw score, which can be compared to the test norms. The internal consistency of the AWST-R was very high, with Cronbach's α = 0.97.

Children's executive functions were measured in the form of behavioral self-regulation both in a subsample of the norm sample and in the FTT sample with the Head Toes Knees Shoulders Task (HTKS) (95). The HTKS was designed for children between the ages of three and six. Children are asked to do the opposite of what the experimenter did (e.g., “If I touch my head, you touch your toes”; “If I touch my toes, you touch your head”). After this first rule was tested in a 10-point block, a second rule and later a third rule were introduced: Touching the shoulders instead of the knees and vice versa. And then a mixture of the first two rules. Compliance with both and later all three rules was tested in a second and third block of 10 trials each. The children's correct responses (two points) and self-corrections (1 point) were tallied (0–60 points).

In the FTT study emotion knowledge was assessed using an abbreviated version of the Test of Emotion Comprehension (TEC) (89) which can be used for children between the ages of 3 and 10. The moral emotion component was omitted because it was too difficult for the 3- to 6-year-olds in the sample. Therefore, the test included 17 items in eight components. Gender-specific versions of the test booklet were available. The TEC contained one to five items for each component. The children looked at the test booklet together with the investigator and were asked to point to the correct emotion from a selection of four emotions. For each component solved, the children received one point, allowing them to score up to eight points. In the FTT sample, Cronbach's α was 0.64.

## Results

### Dimensionality

For the analysis of the dimensionality and the internal consistency of the ATEM 3–9 the software ACER ConQuest (96) was used. The ATEM 3–9 was constructed to measure seven components, that is, seven dimensions of emotion knowledge. Therefore, a seven-dimensional model was computed using the Monte Carlo estimator and 2000 nodes. For this, items 1–6 were assigned to the first component (facial recognition), items 7–12 to the second component (situational recognition), items 13–17 to the third component (desires), items 18–22 to the fourth component (mixed emotions), items 23–27 to the fifth component (beliefs), items 28–32 to the sixth component (display rules), and items 33–35 to the seventh component (emotion regulation strategies). The seven-dimensional model was compared to a unidimensional model computed using Gaussian quadrature. The comparison to a one-dimensional model was chosen to make sure that emotion knowledge captured by the ATEM 3–9 is a multidimensional construct. Only then would an evaluation at the component level make sense. Although there is, for example, the three-factorial model of emotion understanding by Castro et al. (97), the ability domains contained there are not fully covered by the components of the ATEM 3–9. This precludes a comparison with this model. Unlike a factor analysis within the framework of classical test theory, MIRT does not focus on model fit and model comparison, but rather on the individual fit of the items. Nevertheless, it is possible to compare different models with a Chi-square test. A comparison of the seven-dimensional model with the one-dimensional model revealed that the deviance of the seven-dimensional model was significantly lower when compared to the deviance of the one-dimensional model (χ^2^(27, *N* = 882) = 770.54, *p* < 0.001). This suggests that the seven-dimensional model is the better model, which confirms the first hypothesis. Table 4 presents the correlations between the ability distributions of the components of ATEM 3–9 and the variances of the components for this model (latent correlations). The variances of components 3–6 are higher than those of components 1, 2 and 7, because most 3- to 6-year-olds did not score points on the components beyond the termination point after component 2, i.e., all items which follow the drop-out from the test are scored with zero points.

**Table 4:** Latent intercorrelations and variances of the ATEM 3–9 components.

**Component**	**1**	**2**	**3**	**4**	**5**	**6**	**7**
1. Facial recognition							
2. Situational recognition	0.89						
3. Desires	0.92	0.93					
4. Mixed emotions	0.95	0.94	0.94				
5. Beliefs	0.90	0.97	0.93	0.95			
6. Display rules	0.94	0.95	0.96	0.96	0.98		
7. Emotion regulation strategies	0.93	0.88	0.86	0.90	0.91	0.93	
Variance	3.30	1.66	6.84	9.73	11.56	7.78	0.99

### Interitemcorrelations

All 35 items were correlated in the norm sample to identify items that did not share variance with others. According to Cohen (98), correlations with *r* ≥ 0.30 are considered medium and correlations with *r* ≥ 0.50 are considered high. The averaged correlations of the items within a component ranged from *r* = 0.14 (component 6) to *r* = 0.44 (component 4). The mean correlations of the items between components ranged from *r* = 0.16 (between components 1 and 6 and components 6 and 7) to *r* = 0.38 (between components 3 and 4). Almost all items correlated significantly with each other with *r* = 0.07 to *r* = 0.70 (*p* < 0.05). However, items 30 and 31 (in component 6) were significantly correlated with only 12 (34%) other items each. Item 32 (in component 6) correlated significantly with 21 (60%) of the other items.

### Itemfit

**Figure 1:**
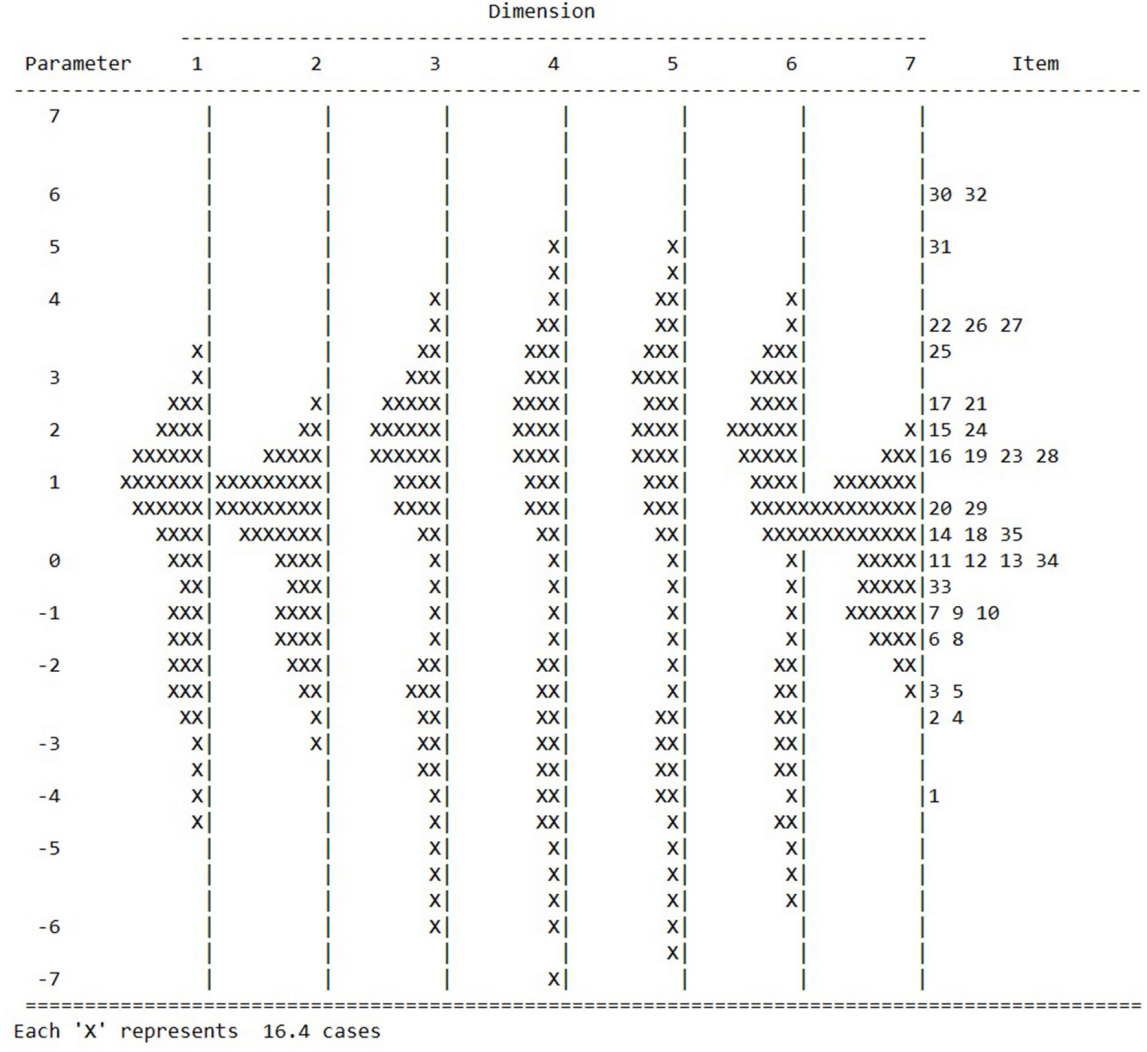
Item- and person parameters of the ATEM 3–6 dimensions.

In a Rasch-scaled test, the item parameters should be distributed over the entire range of person parameters so that the entire range of performance of the tested persons is covered. Figure 1 compares the item and person parameters of the seven-dimensional model. Figure 1 suggests that the item parameters cover the person parameters well. For components 3–6, many children's scores are located in the lower range of the performance spectrum whose performance levels are not covered by test items.

Table 5 lists the individual items of the components of the ATEM 3–9 with their psychometric properties. This includes the item difficulty (*p*), which indicates the proportion of children who solved this item correctly. It is also reported as a standardized item parameter with the associated standard error. At a solution probability of 50%, i.e., an average item difficulty, the item parameter is zero. The smaller the value, the lower the difficulty. According to Fisseni (99), the discriminatory power (*r*__*i*_(t−*i*)_) should be at least 0.30, very discriminatory items are > 0.50. The fit of an item to the Rasch model is estimated with the weighted root mean square (WMNSQ), and the associated t-value. The expected value of the WMNSQ is 1.00. It should not exceed 1.25, whereas a value below 1.00 is not considered to be a problem (100).

**Table 5:** Item characteristics of the ATEM 3–9.

**Component**	**Item**	**Target Emotion**	***N***	***p*-Value**	***r_i(t−i)_***	**Parameter**	***SE***	**WMNSQ**	**t**
Facial recognition	1	Joy	882	0.93	0.38	−3.996	0.156	0.97	−0.3
	2	Anger	882	0.86	0.43	−2.780	0.118	1.08	1.3
	3	Sadness	882	0.82	0.50	−2.370	0.110	1.06	1.0
	4	Surprise	882	0.86	0.49	−2.879	0.120	0.99	−0.1
	5	Disgust	882	0.80	0.52	−2.148	0.107	1.09	1.6
	6	Fear	882	0.74	0.59	−1.629	0.101	1.02	0.4
Sit. recognition	7	Disgust	882	0.72	0.61	−1.186	0.088	0.85	−3.4
	8	Sadness	882	0.78	0.49	−1.626	0.095	0.96	−0.7
	9	Anger	882	0.70	0.51	−1.036	0.087	1.02	0.6
	10	Joy	882	0.69	0.47	−1.000	0.086	1.08	1.8
	11	Fear	882	0.51	0.43	0.023	0.079	1.13	3.7
	12	Surprise	882	0.51	0.54	0.023	0.079	0.98	−0.7
Desires	13	Joy	881	0.58	0.83	−0.361	0.101	0.69	−5.2
	14	Sadness/Disgust	881	0.53	0.78	0.165	0.097	0.81	−3.6
	15	Anger	881	0.32	0.53	1.769	0.092	1.12	3.0
	16	Sadness	881	0.37	0.58	1.371	0.091	1.11	2.7
	17	Joy	881	0.23	0.39	2.509	0.097	1.23	4.7
Mixed emotions	18	Surprise/joy	881	0.53	0.79	0.287	0.105	0.86	−2.3
	19	Sadness/fear	879	0.38	0.66	1.624	0.097	0.99	−0.3
	20	Fear/disgust	878	0.48	0.76	0.696	0.102	0.89	−2.0
	21	Joy/anger	878	0.30	0.60	2.252	0.097	1.01	0.2
	22	Joy/fear	878	0.15	0.39	3.711	0.114	1.16	2.3
Beliefs	23	Sadness	877	0.42	0.67	1.450	0.100	1.09	1.9
	24	Fear	877	0.35	0.60	2.044	0.099	1.20	4.0
	25	Joy/fear	876	0.20	0.50	3.432	0.107	1.06	1.1
	26	Sadness/joy	876	0.16	0.47	3.800	0.113	1.01	0.2
	27	Anger/fear	875	0.18	0.51	3.597	0.110	0.97	−0.6
Display rules	28	Sadness	875	0.36	0.65	1.588	0.093	1.00	0.0
	29	Anger	875	0.47	0.75	0.798	0.097	0.89	−2.3
	30	Joy/fear	874	0.03	0.12	5.652	0.223	1.15	0.9
	31	Anger/joy	872	0.04	0.23	5.094	0.181	1.03	0.2
	32	Sadness/joy	869	0.03	0.18	5.646	0.223	0.98	0.0
Em. reg. strategies	33	Disgust	882	0.62	0.28	−0.570	0.077	1.16	4.5
	34	Fear	882	0.54	0.40	−0.240	0.074	1.05	1.6
	35	Anger	875	0.46	0.73	0.243	0.074	0.73	−10.6

*p, solving probability (difficulty); r_i(t−i)_, discriminatory power; SE, standard error; WMNSQ, weighted mean square; t, test statistic; Sit. recognition, Situational recognition; Em. reg. strategies, Emotion regulation strategies*.

Item 11 (WMNSQ = 1.13, *t* = 3.7), Item 15 (WMNSQ = 1.12, *t* = 3.0), Item 16 (WMNSQ = 1.11, *t* = 2.7), Item 17 (WMNSQ = 1.23, *t* = 4.7), Item 22 (WMNSQ = 1.16, *t* = 2.3), Item 24 (WMNSQ = 1.20, *t* = 4.0), and Item 33 (WMNSQ = 1.16, *t* = 4.5) deviated significantly from the expected value of 1.00 but were below 1.25. The discriminatory power of the items ranged from *r*__*i*_(t−*i*)_ = 0.12 to *r*__*i*_(t−*i*)_ = 0.79. Especially the three very difficult items on Display Rules in component 6 (items 30 to 32) showed an insufficient discriminatory power. Only 3 and 4% of the children, respectively, were able to solve these items correctly.

The skip and termination rules of the components stipulate that the first two components, i.e., the first 14 items (including the two items of component 7 on emotion regulation strategies), should be presented to every child. A linear increase in difficulty within these components to capture children's abilities is therefore not necessary. This requirement is fulfilled nevertheless with one exception each in component 1 (items 3 and 4) and component 2 (items 7 and 8). Within components 3 to 7, the difficulties always increase from the first to the last item of the component with one exception in two adjacent items each (components 3: items 15 and 16; component 4: items 19 and 20; component 5: items 26 and 27; component 6: items 28 and 29).

Table 6 shows the averaged item scores of the components of ATEM 3–9. Item difficulties/item parameters increased from the first to the sixth component. In addition, EAP/ PV reliabilities, a measure of internal consistency which should be interpreted like Cronbach's alpha (101), of the components ranged from 0.78 to 0.91.

**Table 6:** Mean item characteristics for the components of the ATEM 3–9.

**Component**	**Mean item parameter**	**Mean difficulty**	**Mean discrimination**	**EAP/PV reliability**
Facial recognition	−2.634	0.83	0.49	0.87
Situational recognition	−0.800	0.65	0.51	0.88
Desires	1.091	0.41	0.62	0.89
Mixed emotions	1.714	0.37	0.64	0.92
Beliefs	2.865	0.26	0.55	0.90
Display rules	3.756	0.18	0.39	0.91
Emotion regulation strategies	−0.150	0.54	0.47	0.82

*EAP/PV, expected a-posteriori/plausible values*.

### Validity

**Figure 2:**
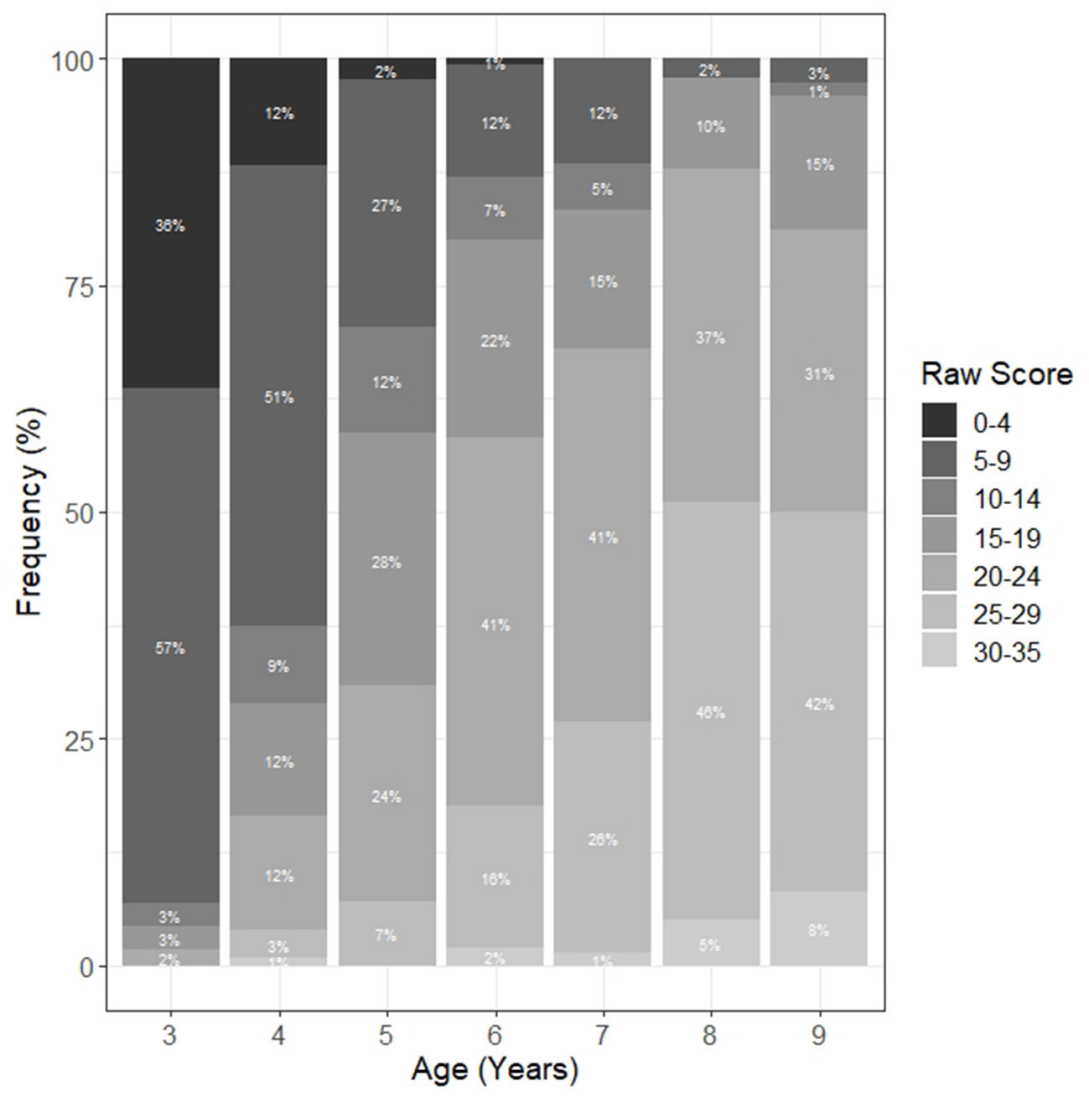
Distribution of the total raw score of the ATEM 3–9 grouped by age.

For the norming procedure, children of the norm sample were divided into seven age groups. Raw scores were calculated at both the level of the total test and the level of the individual components for each of the age groups. The less demanding components 1 and 2 (and component 7) showed rather left-skewed distributions especially among the older children, whereas components 3 to 6 showed extremely right-skewed distributions among the younger children due to the frequent use of the test termination criterion after component 2, which resulted in a score of 0 for all subsequent items. The distribution of the total raw scores of the ATEM 3–9 for the different age groups is depicted in Figure 2. The figure also shows that the values for the younger children assume a rather right-skewed distribution and for the older children a rather left-skewed distribution. In component 6, no child reached the maximum score of five points. Thus, the maximum score of 35 points on the overall test was not reached either. Among the 120 3-year-olds, 111 (96%) terminated the test after component 2 because of the termination rule. For three of the remaining nine children, the skipping rule was applied in component 3 and for four of them in components 4–6. At 4 years of age, 67% of the children terminated the ATEM 3–9 after component 2 and 3% (component 3) to 23% (component 5) of the children who continued the test skipped items of the following components.

Differential item functioning (DIF) analyses were conducted in ConQuest to investigate the effects of children's age, gender, and multilingualism on all component scores and the total score of the ATEM 3–9. The deviances of person parameters from zero for age groups, gender, and multilingualism are displayed in Table 7. Overall, the difference between the person parameters of all adjacent age groups varied between 0.028 (6- and 7-year-olds in the total score) and 1.841 (3- and 4-year-olds in component 4). In summary, the mean performance of the 3-year-olds was lowest in all components and the total test, and that of 8-year-olds was highest in components 2–7 and in the total test. Only in component 1 did the 9-year-olds perform better than the 8-year-olds. In accordance with the age-related differences on the component level, age-related DIF was evident in the majority of items, especially for 3- and 8-year-olds. Alltogether, the results were in line with the second hypothesis.

**Table 7:** Person parameter deviance estimates from zero of age groups, gender, and multilingualism for the components and the total scale of the ATEM 3–9.

	**Component**							**Total**
	**1**	**2**	**3**	**4**	**5**	**6**	**7**	
**Age (years)**
3	−2.206	−1.472	−2.926	−3.087	−2.221	−2.207	−1.320	−2.230
4	−1.073	−0.611	−0.754	−1.246	−0.888	−0.592	−0.317	−0.943
5	0.262	−0.096^a^	0.249	0.062^a^	−0.220	−0.193	−0.051^a^	−0.008^a^
6	0.475	0.389	0.881	0.819	0.372	0.554	0.704	0.619
7	0.652	0.527	0.597	0.733	0.706	0.457	0.325	0.591
8	0.699	0.914	1.147	1.786	1.346	1.093	0.582	1.281
9	1.192	0.349	0.806	0.933	0.904	0.887	0.077^a^	0.690
**Gender**
Male	−0.254	−0.200	−0.294	−0.238	−0.232	−0.142	−0.200	−0.192
Female	0.254	0.200	0.294	0.238	0.232	0.142	0.200	0.192
**Multilingualism**
German	0.110^a^	0.189	0.352	0.514	0.320	0.367	0.214	0.269
Multilingual	−0.110^a^	−0.189	−0.352	−0.514	−0.320	−0.367	−0.214	−0.269

^a^*Person parameter is not significantly different from zero*.

In line with hypothesis 3, girls scored higher on all components and on the total score of the ATEM 3–9 than boys. The lowest deviance of the person parameters from zero was in component 6. However, the estimated deviances were low in all components. Gender-related DIF was found for the items 10, 22, and 26. In two items, boys scored lower than girls. DIF was small with estimated deviances from zero between 0.302 (SE = 0.114) and 0.332 (SE = 0.085)

Because multilingual children scored significantly lower on all components except for component 1, their total score was lower. All estimated deviances from zero were very low in components 1 and 2 and slightly higher in the more complex components, which is in line with hypothesis 4. The highest deviance was found in component 5. DIF for multilingualism was found for eight items (items 3, 8, 11, 14, 16, 19, 20, 33). However, monolingual children scored higher than multilinguals on five of them (items 3, 8, 11, 16, 33) and lower on the other three (item 14, 19, 20). Highest deviance in favor of monolingual children was found for item 3 with 0.540 (SE = 0.110). Highest deviance in favor of multilingual children was found for item 19 with 0.490 (SE = 0.098).

To determine the convergent validity of the ATEM 3–9, the total score and the component scores from the norm sample and the FTT sample were correlated with the total score of the TEC and with the measures of expressive and receptive vocabulary and behavioral self-regulation while controlling for children's age and gender. For these analyses the software R (102) was used. The Pearson correlations are displayed in Table 8. All partial correlations with the ATEM 3–9 total score were significant. Also, all components correlated significantly with the Test of Emotion Comprehension (TEC) in the FTT sample. Especially for younger children, aged three to six, partial correlations with language abilities were medium sized and significant. For the small subsample of older children who took the receptive vocabulary test, the partial correlations were smaller and significant for only a few components. Partial correlations with behavioral self-regulation were rather small in both samples. In summary, these results are in line with hypothesis 5.

**Table 8:** Pearson correlations of ATEM 3–9 scores with other constructs, controlled for age and gender.

**Construct**	**Sample**	**Components of ATEM 3–9**							**t^**a**^**
		**1**	**2**	**3**	**4**	**5**	**6**	**7**	
Emotion knowledge	FTT: *N* = 192 (3–5 y.)	0.31*	0.36*	0.32*	0.24*	0.30*	0.24*	–	0.42*
Sentence understanding	Norm: *N* = 160 (3–6 y.)	0.28*	0.48*	0.37*	0.40*	0.34*	0.36*	0.37*	0.49*
	FTT: *N* = 186 (3–5 y.)	0.43*	0.49*	0.39*	0.26*	0.23	0.25	–	0.52*
Receptive vocabulary	Norm: *N* = 48 (6–9 y.)	0.19	0.33*	0.39*	0.24	0.33*	0.30*	−0.05	0.44*
	FTT: *N* = 179 (3–5 y.)	0.60*	0.49*	0.39*	0.27*	0.26*	0.22*	–	0.58*
Expressive vocabulary	FTT: *N* = 119 (3–5 y.)	0.58*	0.42*	0.51*	0.31*	0.22	0.31	–	0.60*
Behavioral self-regulation	Norm: *N* = 222 (3–6 y.)	0.14	0.19*	0.10	0.16	0.13	0.18*	0.26*	0.22*
	FTT: *N* = 162 (3–5 y.)	0.30*	0.23	0.24	0.21	0.22	0.22	–	0.33*

*p < 0.05.

*t, total scale.*

*^a^For the FTT sample, the total score is composed of the sums of components 1–6*.

Because receptive grammar was strongly associated with multilingualism [*t*_(49)_ = 4.616, *p* < 0.001], analyses of covariance were conducted to examine the effect of multilingualism on emotion knowledge beyond the effect of language abilities. When including the test of receptive grammar (Sentence Understanding) as a covariate, multilingualism showed a trend (*p* < 0.10) to explain additional variance of emotion knowledge for components 3, 4, and 7, and the total test score. For all other components, the effect of multilingualism on emotion knowledge was not significant beyond the effect of receptive grammar.

To calculate the criterion validity of the ATEM 3–9, a matching procedure (nearest neighbor matching) (103) was used to match each child from the psychiatric sample with a maximum of three children from the norm sample. On average, the groups did not differ in age, gender, or multilingualism. Due to missing values, group sizes differed according to the component of the ATEM 3–9 examined. *T*-tests were used to examine whether performance on the ATEM 3–9 differed between the groups. Due to the small size of the psychiatric sample and because it only involved children between the ages of six and nine, age was not included in the analyses. In line with hypothesis 6, there were significant differences in favor of the children from the norm sample in the components Facial Recognition [*t*_(68)_ = 2.189, *p* = 0.032], Mixed Emotions [*t*_(75)_ = 2.578, *p* = 0.012], Beliefs [*t*_(100)_ = 2.776, *p* = 0.007], and Display Rules [*t*_(64)_ = 3.121, *p* = 0.003]. This resulted in a significant difference between the two groups in the total test score [*t*_(62)_ = 2.722, *p* = 0.008]. The difference between the means was rather small at 0.27 in component 1, but at 0.65, 0.65, and 0.51, respectively, in the other components, the differences were greater than half a point.

## Discussion

The ATEM 3–9 was developed in accordance with IRT with the aim of assessing the emotion knowledge of children between 3 and 9 years of age. It measures seven components of emotion knowledge of increasing complexity in an adaptive design, which corresponds to the development of emotion knowledge in childhood (2). In the present work, the dimensional structure of the ATEM 3–9, the psychometric properties of the test items and the total test were examined as well as indicators of its reliability and validity.

Analyses within the framework of MIRT with test scores collected from *N* = 882 children showed that the seven-dimensional model of the ATEM 3–9 was superior to the unidimensional model of overall emotion knowledge. This result was in line with the first hypothesis. It confirms the hypothesis that emotion knowledge is composed of different components. The rather high latent correlations between the seven components suggest that they are not independent from each other but that they also do not measure the same dimension. High correlations rather indicate a high efficiency of the ATEM 3–9, because it is possible to obtain much information with few items, compared to a unidimensional solution for each component (104). High efficiency is also demonstrated by the good to very good reliability estimates of the components which could not have been achieved with several unidimensional analyses with so few items. With the ATEM 3–9 it is therefore possible to reliably examine children's performance on seven components of emotion knowledge with only one instrument. This is a unique characteristic of the test compared to tests like the TEC (89) or the Affective Knowledge Task (73, 105). Whereas a total score on the ATEM 3–9 means that the child's emotion knowledge is developed in an age-appropriate manner overall, it could be that above-average scores in one component of emotion knowledge are compensated by below-average scores in another component. Therefore, it is always recommended to interpret the results at the component level as well. Below-average scores in individual components point to areas which are possibly in need of intervention, while above-average scores in individual components can be considered resources.

In addition to some overlap in content, the mean partial correlation with the TEC as another measure of emotion knowledge indicates that the ATEM 3–9 does not measure exactly the same construct as the TEC. Given the only partially overlapping components of the ATEM 3–9 and the TEC, this is consistent with our expectations in hypothesis 5.

The increasing variances of the component ability distributions underline ATEM 3–9's ability to discriminate between individuals in the more complex components. Many of the younger children were able to solve only a minority of the items in components 3–6 or terminated the test after component 2 due to the termination rules. This is also evident in the distribution of the person- and item parameters in Figure 1, which showed that many persons in the lower range of the performance spectrum in component 3–6 were not covered by the test. The distribution of raw scores in Figure 2 corroborates this state of affairs. The fact that only 4% of the 3-year-olds and 33% of the 4-year-olds reached components 3–6 confirms that using the termination and skip rules was reasonable.

The ATEM 3–9's characteristics on the item and the component level confirm that the intended structure with increasing difficulties within and between components was successfully implemented. The few exceptions were not important for an accurate description of a child's ability because the solution of these items did not affect the application of skip- or termination rules. No item exceeded the expected value of the WMNSQ of 1 to such an extent that the fit of the item to the Rasch model could no longer be assumed. In addition, the discriminatory power of the items was good, except for three items of component 6.

Three of the items in component 6 correlated with only 34 and 60% of the other items. As the item characteristics show, these items were solved correctly by only 3 and 4% of the children and thus had insufficient discriminatory power. The text load of these items is very high, and children must remember much complex information. The correlations of component 6 with behavioral self-regulation, which includes aspects of working memory, was rather high, when compared to the correlations of behavioral self-regulation with the other components. Thus, the reason for the high difficulty (or low probability of solving these items) may have been not so much the lack of children's emotion knowledge, but rather the complexity of the items and their demands on working memory. However, emotion knowledge is not completely detached from working memory. Even in everyday social interactions much information must be processed in working memory in order to be able to make person- and situation-specific statements about their emotional states. Executive functions should rather be regarded as an integral part of emotion knowledge. This is also supported by results from Morra et al. (22) who found that the effect of age dropped below significance when working memory was entered as a predictor of emotion knowledge. Older children with more developed working memory capacities may be better able to solve the more difficult items of component 6. Rather than violating the fixed structure of the ATEM 3–9 by excluding these items, the heavy demands on working memory suggest that extending the age range of the ATEM 3–9 to 10- and 11-year-olds may be appropriate.

An additional argument for an extension of the age range lies in the fact that so far no child in the present sample of over 800 children has reached the full score of 35 points in the ATEM 3–9. Nevertheless, as expected, children's performance on the ATEM 3–9 improved with age, which supports our second hypothesis and previous studies on emotion knowledge (2, 58, 106). The 5-year-olds showed the smallest deviations of the person parameters from the mean (from zero). Differences between neighboring age groups were small but significant in most cases. Providing different norm tables of the ATEM 3–9 for the different age groups is therefore necessary.

Male and female children differed significantly at the component level and at the total score. Differences were the smallest in component 6 and at the level of the total score. This meets our expectations in hypothesis 3 and confirms previous research on gender differences in emotion knowledge, which concludes that there are no or minimal differences in general emotion knowledge but remarkable differences in individual components (4, 22, 60, 62, 67). However, many of these gender differences depended partly on age (4). Because small groups resulted when dividing children into different age- and gender-groups, separate norm tables for boys and girls could not be realized. However, gender differences should be kept in mind, when interpreting and comparing ATEM 3–9 scores.

The performance of children growing up in multilingual families did not differ from the performance of children growing up in monolingual homes in component 1, i.e., Facial Recognition. This is the component which contains the least amount of text and therefore places the least demands on children's receptive language skills. In line with hypothesis 4 , the difference between the monolingual and multilingual group were the greatest in component 4. When Sentence Understanding was included in analyses of covariance as a covariate, growing up with multiple languages contributed to the explanation of additional variance of emotion knowledge only for some components as a trend. These results suggest that less developed language skills may be more important predictors of children's emotion knowledge than cultural aspects of multilingualism, such as having one or two parents who were born abroad. In order to account for these differences, separate norm tables for monolingual children on the one hand and monolingual and multilingual children on the other hand were generated for the ATEM 3–9. Due to the small proportion of multilingual children in the norm sample, it was not possible to create separate norm tables for these children. When interpreting the results in the ATEM 3–9 from multilingual children, the mixed norm table will give somewhat less strict cutoff values. Conversely, monolingual children are rated more strictly because possible effects of language skills that attenuate cutoff scores are accounted for in the norm table for monolingual children. However, when assessing children's emotion knowledge with the ATEM 3–9 and interpreting the results, language abilities should be kept in mind as a possible influence.

When comparing the performance of children with a mental disorder with their typically developing agemates the former tended to show a less developed emotion knowledge than the latter. This is consistent both with hypothesis 6 and with previous findings that the emotional competencies of children with various mental disorders are often not developed in an age-appropriate manner (38, 42, 107). This result provides not only evidence for the criterion validity of the ATEM 3–9, but also demonstrates the value of the ATEM 3–9 for clinical practice, where instruments for reliably assessing the individual components of emotion knowledge – particularly the more sophisticated ones – have not been available so far.

### Limitations

In addition to the many advantages of the ATEM 3–9, some limitations must be noted. On the side of the study, larger groups of children of different ages and sociodemographic characteristics should be addressed in future research to gain more information of, for example, gender differences in emotion knowledge for children of different ages. In addition, the question of whether the ATEM 3–9 is appropriate for 10- and 11-year-olds remained open, because no ceiling effects appeared in the performances. More data should also be obtained on immigrant children or on children growing up in multilingual families to account for their less developed language abilities in norm data. Clinical samples should be divided by specific disorders before comparing the children in treatment with their typically developing agemates. This was not possible in the present sample due to the small sample size. Thus, larger clinical samples are needed. On the side of the ATEM 3–9, some items in component 6 seemed to be too difficult for most children, even for the oldest ones. In a future revision of the ATEM 3–9, assessing the knowledge of emotional display rules should be carried out in a manner that requires less working memory capacity. This can be done, for example, by condensing the text of the items even further. Items which showed DIF regarding gender or multilingualism should also be revised and adjusted, for example, by changing response options.

### Areas of Application of the ATEM 3–9

The ATEM 3–9 was developed to measure children's emotion knowledge from the perspective of developmental psychology and psychopathology. The reliable assessment of individual components makes the ATEM 3–9 well suited for profile analyses which illustrate well-developed and poorly developed domains of emotion knowledge. This can be helpful when trying to identify a child's emotional resources. The ATEM 3–9 is also well suited for monitoring the development of emotion knowledge, after an intervention or prevention program, a social training, a mindfulness intervention, or during psychotherapy. The assessment of emotion knowledge is also particularly interesting in the case of mental disorders in children [e.g., attention deficit (hyperactivity) disorder, autistic spectrum disorders, social behavior disorder, affective disorders, etc.] as well as in the case of developmental delays in emotional competencies. Because children's emotion knowledge contributes to the explanation of their academic skills (36), using the ATEM 3–9 in educational-psychological research and practice is also conceivable. Because the ATEM 3–9 is available in German, English, and Hebrew it can also be used in cross-cultural comparisons. Moreover, in a small sample of *N* = 21 children who performed the ATEM 3–9 *via* tele-assessment, 86% reported that they enjoyed completing the ATEM 3–9 (108). This is consistent with the positive feedback that was provided by many children of the norm sample and the FTT sample.

## Conclusion

In summary, in the present study with norm data from Germany, the ATEM 3–9 has a seven-dimensional structure which aligns with the seven components of emotion knowledge that it aims to measure. Most of the 35 items showed a good item fit with increasing item difficulties within and between the components. Revising three very difficult items in the component that assesses the knowledge about display rules may be reasonable. Future research should also examine whether the ATEM 3–9 is also appropriate for older children. Because the ATEM 3–9 is the first instrument which reliably depicts the development of individual components of children's emotion knowledge it seems to be not only valuable as a performance test of an important component of emotional competence but also useful for clinical research and practice.

## Data Availability Statement

The raw data supporting the conclusions of this article will be made available by the authors, without undue reservation.

## Ethics Statement

The studies involving human participants were reviewed and approved by Ethics Committee of Leuphana University Lüneburg. Written informed consent to participate in this study was provided by the participants' legal guardian/next of kin.

## Author Contributions

KV and MS contributed to the design of the study, manuscript revision, read, and approved the submitted version. KV organized the database and performed statistical analyses and wrote the first draft of the article.

## Funding

ATEM 3–9 data were collected in the multicenter Feeling Thinking Talking study which was funded by the German Federal Ministry for Family, Senior Citizens, Women, and Youth under the grant BIS00.00007.16.

## Conflict of Interest

The authors declare that the research was conducted in the absence of any commercial or financial relationships that could be construed as a potential conflict of interest.

## Publisher's Note

All claims expressed in this article are solely those of the authors and do not necessarily represent those of their affiliated organizations, or those of the publisher, the editors and the reviewers. Any product that may be evaluated in this article, or claim that may be made by its manufacturer, is not guaranteed or endorsed by the publisher.
